# Identification and Outcomes of Hospitalized Medically Ill Patients Who Are Candidates for Extended Duration Thromboprophylaxis

**DOI:** 10.1055/s-0040-1718911

**Published:** 2020-10-31

**Authors:** Craig I. Coleman, Gregory Piazza, Veronica Ashton, Thomas J. Bunz, Alex C. Spyropoulos

**Affiliations:** 1 Department of Pharmacy Practice, University of Connecticut School of Pharmacy, Storrs, Connecticut, United States; 2 Evidence-Based Practice Center, Hartford Hospital, Hartford, Connecticut, United States; 3 Division of Cardiovascular Medicine, Brigham and Women's Hospital, Boston, Massachusetts, United States; 4 Harvard Medical School, Boston, Massachusetts, United States; 5 Real World Value & Evidence, Janssen Scientific Affairs, LLC, Titusville, New Jersey, United States; 6 Division of Pharmacoepidemiology, New England Health Analytics, LLC, Granby, Connecticut, United States; 7 Institute for Health Innovations and Outcomes Research, Feinstein Institutes for Medical Research and the Zucker School of Medicine at Hofstra/Northwell, New York, New York, United States; 8 Department of Medicine, Anticoagulation and Clinical Thrombosis Services, Northwell Health at Lenox Hill Hospital, New York, New York, United States

**Keywords:** anticoagulation, extended duration thromboprophylaxis, medically ill, venous thromboembolism, prevention

## Abstract

**Background** Extended duration thromboprophylaxis (ET) for approximately 30 days can effectively and safely reduce venous thromboembolism (VTE) risk in appropriately selected medically ill patients. We sought to estimate the proportion of hospitalized medically ill patients potentially qualifying for ET and assess their post-discharge clinical and economic outcomes using a large claims database.

**Methods** Using MarketScan claims from January 2012 to September 2018, we identified medically ill patients hospitalized with a primary diagnosis of heart failure, respiratory insufficiency, ischemic stroke, infection, or inflammatory disease and ≥1-additional risk factor for VTE. Patients < 40 years old, a length-of-stay < 3 or >30 days, receiving oral anticoagulation prior to index hospitalization or having an indication for full-dose anticoagulation were excluded, as were patients deemed high-risk for bleeding due to active, in-hospital treated cancer, gastroduodenal ulcer or bleeding within the prior 3 months, bronchiectasis, pulmonary cavitation or hemorrhage, or dual antiplatelet therapy use.

**Results** We identified 2,782,988 patients ≥40 years of age and admitted for a high-risk medical illness. Of these, 724,531 patients (26.0%) were identified as ET candidates. Patients' VTE risk appeared highest in the first 30 days post-discharge (1,532/724,531, 0.2%). Adjusted post-index hospitalization costs (2018 US$) for patients with a VTE within 30 days were higher than those without VTE (Δ = $32,623 at 30 days, Δ = $43,325 at 90 days, Δ = $53,668 at 365 days; *p* < 0.001 for all).

**Conclusion** Post-discharge VTE in high-risk patients with medical illness is associated with substantially increased costs.

## Introduction

Patients with an acute medical illness such as heart failure, respiratory insufficiency, ischemic stroke, infection, or inflammatory disease are highly susceptible to the development of venous thromboembolism (VTE) during their hospital stay and up to 3 months post-discharge, with the highest risk period during the first month. 1 2 Randomized controlled trial data have demonstrated extended duration thromboprophylaxis (ET) with direct oral anticoagulants for approximately 30 days to prevent VTE which may be associated with a favorable benefit-risk profile when administered to carefully selected patients at high-risk for VTE but lower risk of bleeding. 3 4 5 6 However, there is a relative paucity of studies estimating the proportion of patients that could be considered candidates for ET and assessing their post-discharge incidence of VTE, health care utilization, and costs. In the present study, we sought to estimate the proportion of hospitalized medically ill patients potentially qualifying for ET and assess their post-discharge clinical and economic outcomes using a large claims database.

## Methods

We performed a retrospective analysis using United States IBM MarketScan claims data from January 1, 2012 through September 30, 2018. IBM MarketScan combines two separate databases, a commercial claims and encounters (CCAE) and a Medicare supplemental database, to cover all age groups; and contains claims from approximately 260 contributing employers, approximately 40 health plans and government and public organizations representing approximately 240 million lives. 7 IBM MarketScan captures enrollment records, demographics, International Classification of Diseases, Ninth- and Tenth-Revision (ICD-9 and ICD-10) diagnosis codes, procedure codes, admission and discharge dates, outpatient medical services data, and prescription dispensing records. All IBM MarketScan data are de-identified and are thus in compliance with the Health Insurance Portability and Accountability Act of 1996 to preserve patient anonymity and confidentiality. This study was determined to not constitute research involving human subjects according to 45 Code of Federal Regulations 46.102(f) and deemed exempt from board oversight.

### Patient Identification

This study defined candidacy for ET as a hospitalization for a medical illness deemed to place a patient at higher risk for developing a VTE but at a lower risk of bleeding according to the criteria utilized in the Multicenter, Randomized, Parallel Group Efficacy and Safety Study for the Prevention of Venous Thromboembolism in Hospitalized Acutely Ill Medical Patients Comparing Rivaroxaban with Enoxaparin (MAGELLAN) subpopulation study. 3 This study evaluated rivaroxaban versus enoxaparin/placebo for 35 ± 4 days to prevent either symptomatic or asymptomatic thrombotic events. The MAGELLAN subpopulation criteria for ET were chosen *a priori* for this study because they are more easily implemented in claims databases as compared with criteria utilized in other ET trials which depend on knowledge of D-dimer levels (which were not available in our MarketScan dataset). 3 4 5 6

To be included in this study, patients had to be hospitalized with a primary discharge diagnosis code for heart failure, respiratory insufficiency, ischemic stroke, infection, or inflammatory (including rheumatic) disease. Patients <40 years old, having a length-of-stay (LOS) <3 or >30 days, dying prior to discharge, having stage 4 or worse chronic kidney disease, receiving oral anticoagulation therapy prior to the index hospitalization or having an indication for full-dose anticoagulation at baseline were excluded. Medically ill patients also had to have at least one additional risk factor for VTE including prolonged immobilization, ≥75 years, morbid obesity, a past medical history of cancer, heart failure or thrombophilia, a personal history of VTE (ICD-10 code of Z86.718 or ICD-9 code of V12. 51 only) or an acute infectious disease contributing to the index hospitalization (the latter depicted by the presence of a nonprimary discharge diagnosis code for any infection). 3 As done in a prior study, 8 a LOS ≥3 days was considered a proxy for prolonged immobilization. Finally, to identify a cohort of patients at lower risk of bleeding as was done in the MAGELLAN subpopulation analysis, 3 we excluded patients taking dual antiplatelet therapy or those with active, in-hospital treated cancer, gastroduodenal ulcer or bleeding within the prior 3 months, bronchiectasis, pulmonary cavitation, or hemorrhage.

### Outcome Assessment

Upon the identification of medically ill patients that were candidates for ET, we performed a descriptive analysis to assess patient demographics, baseline comorbidities, index hospitalization characteristics, and post-index hospital discharge outcomes.

The primary outcome of interest was the incidence of 30-day post-discharge VTE. 9 10 VTE was defined as a hospitalization with a primary discharge diagnosis code for either deep vein thrombosis and/or pulmonary embolism (PE); an emergency department or observation unit encounter with a primary diagnosis code for DVT and/or PE (without a DVT or PE code in the prior 12 months) and accompanied by both a billing code for a VTE diagnostic test (e.g., computed tomography, magnetic resonance imaging, ventilation–perfusion scan, ultrasound) and new initiation of full-dose anticoagulation; or an outpatient visit with a diagnosis code for DVT and/or PE in any position (without a DVT and/or PE code in the prior 12 months) and accompanied by both a billing code for a VTE diagnostic test and new initiation of full-dose anticoagulation. Secondary clinical outcomes included time-to-post-discharge VTE, the incidence of rehospitalization for any cause and incidence of a post-index hospitalization major adverse cardiovascular event (defined as a subsequent hospitalization with a primary discharge diagnosis code for myocardial infarction or stroke) at 30 days post-index hospitalization, as well as the incidence of recurrent VTE (second post-discharge VTE defined as a hospitalization with a primary diagnosis code for DVT and/or PE) at 365 days post-discharge.

The primary economic outcome for this study was total post-hospital discharge costs, including inpatient, outpatient, and outpatient pharmacy costs (but not including the costs of the index hospitalization). Costs were inflated to 2018US$ using the consumer price index for Medical Care available from the Bureau of Labor Statistics. 11 We compared total costs at 30, 90 and 365 days between patients who developed a post-index hospitalization VTE within 30 days of discharge (early VTE) and those who did not.

Subgroup analysis was performed in which we evaluated the incidence of VTE and total post-hospital discharge health care costs in patients with an additional risk factor for VTE that was present in >5% of the ET candidate population (i.e., ≥75 years old, morbid obesity, history of heart failure, history of cancer).

Continuous data were summarized as means ± standard deviations (SDs). Categorical outcomes were reported as incidences ( *n* , %); and Kaplan-Meier analysis was performed to assess time-to-first post-index hospitalization VTE. Testing for statistical significance of unadjusted total post-hospital discharge costs between those developing and not developing early VTE was performed using an independent samples *t* -test. Adjusted differences in total post-hospital discharge costs between those developing and not developing early VTE were also estimated using generalized linear regression models including sex, primary reason for the index hospitalization, and 12 comorbidities/risk factors for VTE including history of cancer, history of VTE, history of heart failure, thrombophilia, morbid obesity, acute infectious disease contributing to admission, prolonged immobilization, chronic venous insufficiency, varicosis, recent major surgery or trauma in the prior 6 to 12 weeks, estrogen therapy, and chronic kidney disease.. All data management and statistical analysis were performed using SAS version 9.4 (SAS Institute Inc., Cary, North Carolina, United States) and IBM SPSS Statistics version 25.0 (IBM Corp., Armonk, New York, United States).

This report was written to comply with the Reporting of Studies Conducted using Observational Routinely Collected Health Data (RECORD) statement. 12

## Results

**Fig. 1:**
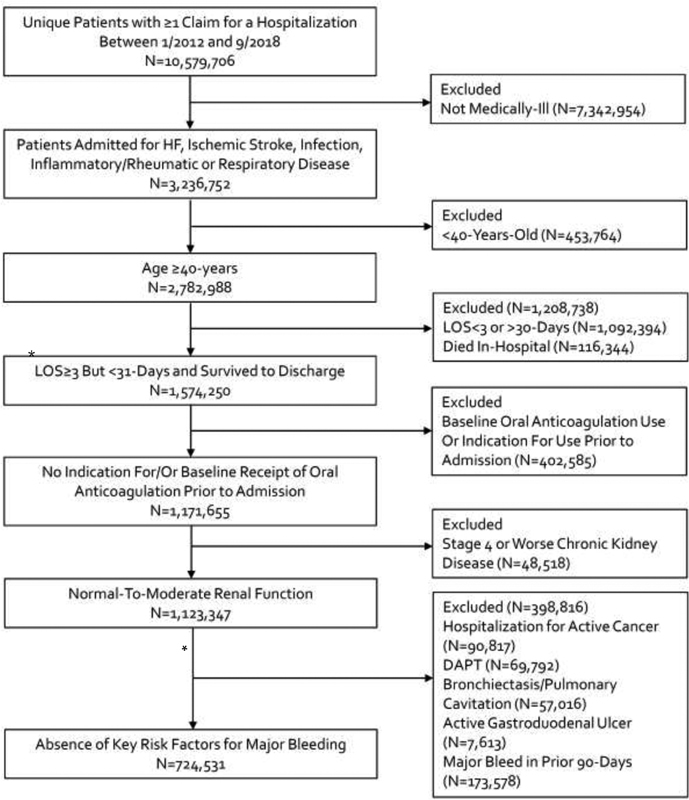
Inclusion and exclusion of patients. DAPT, dual antiplatelet therapy; HF, heart failure; LOS, length-of-stay; *N* , number. *Length-of-stay ≥3-days inclusion criteria was a proxy for prolonged immobilization; and therefore, all patients had at least one additional risk factor for the development of venous thromboembolism

In total, 10,579,706 unique hospitalized medically ill patients admitted for any reason were included in the MarketScan dataset between January 1, 2012 and September 30, 2018. Of these, 3,236,752 were admitted for heart failure, ischemic stroke, infection, inflammatory/rheumatic, or respiratory disease based upon primary hospital billing codes. Age < 40 years resulted in the exclusion of 453,764 patients leaving 2,782,988 patients. After excluding patients with LOS < 3 or >30 days or dying in-hospital, those already taking or having an indication for oral anticoagulation and those with stage 4 or worse chronic kidney disease, 1,123,347 patients remained eligible. From these, 398,816 were excluded for having at least one of the five major bleeding risk factors identified in the MAGELLAN subpopulation, 3 leaving 724,531 patients (26.0% of 2,782,988) as ET candidates. A detailed flow diagram of patient inclusion and exclusion is provided as Fig. 1 .

Characteristics of 724,531 ET candidate patients are described in Table 1 . The most frequent qualifying primary coded cause for the medically ill hospitalization was acute inflammatory or rheumatic disease (38.2%), followed by acute respiratory disease (26.4%), and infection (21.5%), with acute ischemic stroke and heart failure each making up less than one-tenth of the final study cohort. Other than prolonged immobilization, age ≥75 years, prior history of cancer, heart failure, and morbid obesity were the additional VTE risk factors that had a prevalence >5%. The index hospitalization LOS averaged 5.4 ± 3.9 days and cost a mean of $33,290 ± 43,503 per patient.

**Table 1:** Preindex hospitalization characteristics of included patients

Characteristic	*N* = 724,531 *n* (%)
Sex (male)	405,036 (55.9)
Age (y), mean ± standard deviation	64.6 ± 13.3
< 65 y	421,739 (58.2)
65–74 y	126,368 (17.4)
≥ 75 y	176,424 (24.4)
**Reason for hospitalization**	
Heart failure	48,099 (6.6)
Acute ischemic stroke	53,140 (7.3)
Acute infectious disease	155,794 (21.5)
Acute inflammatory or rheumatic disease	276,578 (38.2)
Acute respiratory disease	190,920 (26.4)
**Additional risk factors for venous thromboembolism**	
History of cancer	116,403 (16.1)
History of venous thromboembolism	1,432 (0.2)
History of heart failure	61,052 (8.4)
Thrombophilia	1,056 (0.1)
Age ≥ 75 y	176,424 (24.4)
Morbid obesity	39,848 (5.5)
Acute infectious disease contributing to admission	32,282 (4.5)
Prolonged immobilization a	724,531 (100)
Chronic venous insufficiency or varicosis	14,292 (2.0)
Recent major surgery or trauma (6–12 wk)	10,178 (1.4)
Estrogen therapy	27,262 (3.8)
Chronic kidney disease, stage 3	22,708 (3.1)

a Assumed based on minimum length-of-stay of at least 3 d.

**Fig. 2:**
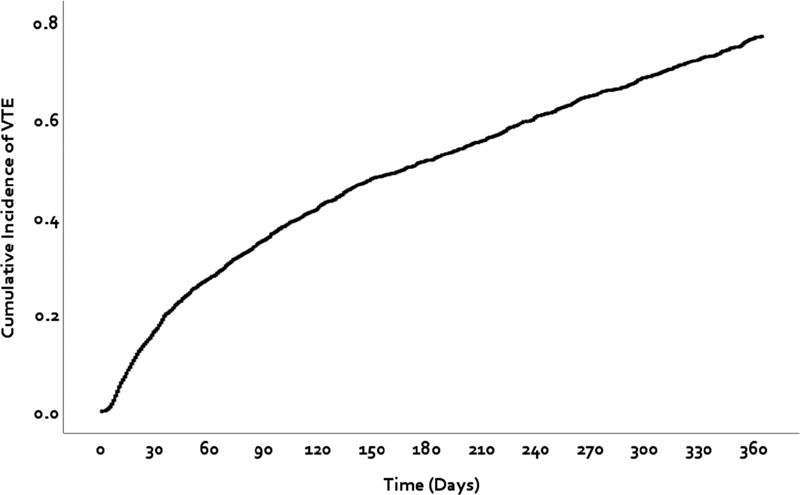
Kaplan-Meier curve for time-to-first venous thromboembolism. VTE, venous thromboembolism.

**Fig. 3:**
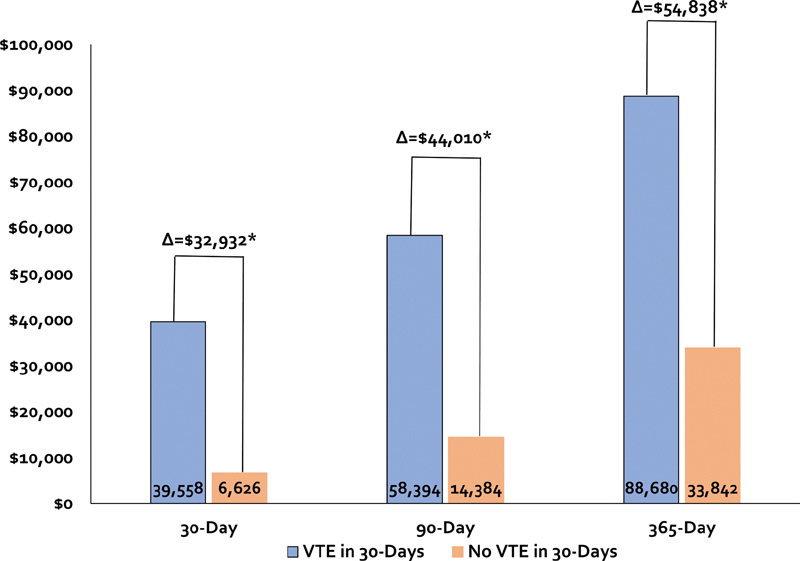
Unadjusted change in 30-, 90- and 365-day costs between medically ill patients experiencing and not experiencing a venous thromboembolism in the first 30-days post-index hospitalization. Δ, delta (difference in costs); VTE, venous thromboembolism. * *p* < 0.001.

The incidence rate of VTE was greatest in the first 30-days post-discharge with 1,532 (0.2%) patients experiencing a VTE ( Fig. 2 ). Total post-hospital discharge health care costs for patients experiencing an early VTE within 30-days of discharge were higher at 30 ($39,558 ± 73,670 vs. $6,626 ± 22,668), 90 ($58,394 ± 101,396 vs. $14,384 ± 39,964), and 365 days ($88,680 ± 131,225 vs. $33,842 ± 78,304) post-index hospitalization than for patients not experiencing a VTE within 30 days. Unadjusted differences in total costs between those with and without a 30 day post-discharge VTE were $32,932 ( *p* < 0.001) at 30 days, increased to $44,010 ( *p* < 0.001) at 90 days, and continued to widen at 365 days ($54,838, *p* < 0.0001) ( Fig. 3 ). Cost differences between patients with and without a 30 day post-discharge VTE remained significantly different after adjustment using generalized linear modeling ($32,623 at 30 days; $43,325 at 90 days, and $53,668 at 365 days; *p* < 0.001 for all).

The incidence of post-discharge VTE and total downstream health care costs in the subset of patients who were ≥75 years old, morbidly obese, or had a history of heart failure or cancer were comparable to the overall ET candidate population ( Table 2 ).

**Table 2:** Post-index hospitalization outcomes in subsets of patients with >5% prevalence of an additional risk factor for venous thromboembolism

Outcome	All patients *N* = 724,531	Morbid obesity *N* = 39,848	≥75 y *N* = 176,424	Heart failure *N* = 61,052	History of cancer *N* = 116,403
**Venous thromboembolism, n (%)**					
30 d	1,532 (0.2)	139 (0.3)	141 (0.1)	89 (0.1)	242 (0.2)
90 d	2,727 (0.4)	251 (0.6)	310 (0.2)	194 (0.3)	472 (0.4)
365 d	4,487 (0.6)	358 (0.9)	798 (0.5)	392 (0.6)	856 (0.7)
**Total costs (2018 US$)**					
30 d	$6,699 ± 22,959	$7,724 ± 26,620	$5,609 ± 14,785	$10,071 ± 31,543	$8,473 ± 25,587
90 d	$14,480 ± 40,253	$17,065 ± 44,877	$11,850 ± 26,162	$23,554 ± 61,126	$18,796 ± 45,577
365 d	$33,962 ± 78,499	$40,345 ± 84,370	$28,848 ± 58,526	$53,400 ± 118,202	$43,475 ± 92,928

US$, United States dollars.

The incidence rates of secondary outcomes at 30 days were 50,049 (6.9%) for rehospitalization for any cause and 1,180 (0.2%) for a major adverse cardiovascular event hospitalization. A second, or recurrent, VTE occurred in 163 of the 4,487 (3.6%) medically ill patients who developed an initial VTE post-discharge during the 365-day post-discharge period.

## Discussion

The present study found that 26% of medically ill patients ≥40 years of age hospitalized in routine practice in the United States would qualify for ET with an approved direct oral anticoagulant such as rivaroxaban according to prescribing labeling. 13 The evaluated medically ill cohort was at high-risk of post-discharge VTE due to patients' primary reason for hospitalization, but at a lower risk of bleeding due to the exclusion of patients with key bleeding risk factors. 3 In the first 30-days post-discharge, 1,532 of 724,531 (or 0.2%) of evaluated medically ill patients were diagnosed with an acute VTE. Patients experiencing a VTE in the first 30-days had significantly higher total post-hospital discharge health care costs than patients who did not experience a VTE, with substantial cost differences seen early (surpassing $32,000 at 30 days, *p* < 0.001) and approaching $55,000 by 1 year ( *p* < 0.0001).

The incidence rate of VTE in our study was estimated to be 0.2% at 30 days and 0.6% at 365-days post-discharge. When interpreting our incidence rates several factors need to be considered. Compared with randomized trials 3 4 5 6 and some real-world studies evaluating “hospital-acquired” VTE and its prevention, 10 14 our study did not count thrombotic events that occurred during the index hospital admission. We opted to not attempt to capture these events as the positive predictive value of identifying acute VTE using nonprimary billing codes (which by default was the case in the present study as we required heart failure, respiratory insufficiency, ischemic stroke, infection, or inflammatory disease to be the primary coded cause) is poor, ranging only between 50 and 75%. 9 Moreover, randomized trials actively sought to identify asymptomatic VTE which often made up >75% of identified cases. 3 In our real-world dataset, it was unlikely that asymptomatic VTE was commonly assessed or identified. Of note, our reported rate of VTE at 30-days was generally consistent with the incidence of symptomatic VTE reported in the MAGELLAN subpopulation at 35 ± 4 days (0.7%) minus those events occurring during the 10 ± 4 days index hospitalization period (0.5%). 3 Finally, as IBM MarketScan data 7 consists largely of commercially insured patients augmented by patients ≥65 years-old with Medicare supplemental insurance, the medically ill cohort evaluated in our study is likely younger and healthier than seen in prior studies. 3 4 5 6 10 For example, while approximately 40% of patients included in the MAGELLAN subpopulation 3 were ≥75 years old, only approximately 25% of patients were of that age in our study. Moreover, our study included a lower proportion of patients with heart failure (8.4%) compared with the MAGELLAN subpopulation (approximately 37%). 3

Prior real-world studies have attempted to estimate the proportion of acute medically ill patients that might benefit from ET. 8 10 A study by Rosenberg et al 10 applied the International Medical Prevention Registry on Venous Thromboembolism (IMPROVE) VTE risk score to a cohort of 19,217 medically ill patients hospitalized within a tertiary health system. They found that 32% of the medically ill patients assessed would be considered high-risk for “hospital-acquired” VTE based on an IMPROVE VTE score ≥ 3. A second study by Miao et al 8 applied the Medically Ill Patient Assessment of Rivaroxaban versus Placebo in Reducing Post-Discharge Venous ThromboEmbolism Risk (MARINER) trial 6 criteria to the 2014 National Inpatient Sample (NIS) dataset to determine the proportion of medially ill patients that might be candidates for ET. Of the 1,849,535 hospitalizations for medical illness captured in the 2014 NIS, 407,095 hospitalizations (22.0%) were found to be in patients at high risk for VTE (per a modified IMPROVE VTE risk score used in MARINER). These prior studies, 8 10 taken together with the present, suggest that between 1 in 5 and 1 in 3 medically ill patients could be candidates for ET. Yet it is estimated that fewer than 5% of medically ill patients receive ET. 15 Guidance from international/national medical societies and standards setting accrediting bodies (e.g., The Joint Commission) improved VTE risk assessment models, decision support tools integrated into electronic health records, and other quality improvement initiatives may be helpful in assuring appropriate (i.e., lower bleeding risk) at-risk medically ill patients are considered for ET.

Our study has several limitations that merit discussion. First, biases such as misclassification can negatively impact the internal validity of claims database analyses such as ours. 14 Second, IBM MarketScan does not contain specific data on immobility. 7 Trials have defined immobility as being confined to a bed or chair for most of the day, with independent mobility only to the in-room toilet for at least 24 hours. 4 5 However, outside of a prospective trial, the assessment of prolonged immobility can be difficult and/or subjective. 16 In our study, we assumed an index hospital LOS ≥3 days was a reasonable proxy for prolonged immobilization. 8 This assumption is supported by a study by Amin and colleagues 17 which identified extended hospital LOS in medically ill patients as a strong risk factor for subsequent VTE development at 6 months (with LOS of 4–6 and ≥7 days associated with a 41 and 221% increased risk of VTE compared with a LOS of 1–3 days). However, it is possible our use of LOS as a proxy for prolonged immobility resulted in an overestimation of patient risk. Third, we classified a medically ill patient's reason for hospitalization based upon their primary billing code for that hospitalization (the sum of the proportion of reasons for hospitalization total to 100%) making direct comparison of our demographic data to that of prior randomized trials 3 4 5 6 difficult. It is likely that many patients in our study had more than one diagnosis (i.e., heart failure, respiratory insufficiency, ischemic stroke, infection, or inflammatory disease) and/or risk factor qualifying them for ET. Fourth, although we adjusted for demographics, primary reason for the hospitalization and 12 key comorbidities/risk factors for VTE in our regression analyses estimating differences in post-index hospitalization costs between patients experiencing and not experiencing an early VTE, residual confounding cannot be ruled out. Moreover, our analysis does not provide an estimate of the proportion of the difference in costs that are directly attributable to the management of VTE or its complications. Finally, we performed this analysis in the IBM MarketScan CCAE and Medicare supplemental databases. While this combined dataset covers many lives spanning all age groups, 7 it is skewed toward a younger and healthier medically ill population (particularly compared with randomized trials) which may have resulted in an underestimation of the proportion of patients eligible for ET in the real world. The use of MarketScan data also makes our findings less generalizable to non-U.S. patients.

## Conclusion

Our study found that 26% of the medically ill patients evaluated met the MAGELLAN subpopulation criteria for use of rivaroxaban for ET. Early post-discharge VTE occurrence in these patients was associated with substantially increased total downstream health care costs. Steps to reduce patients' risk of developing hospital acquired VTE after discharge should be implemented using ET with approved direct oral anticoagulants.
